# Beyond β-amyloid: transthyretin cerebral amyloid angiopathy

**DOI:** 10.1007/s10072-026-09398-2

**Published:** 2026-09-26

**Authors:** Carla Vera-Cáceres, Carolina Montecino Romanini, Montserrat González Platas, Irene Sánchez-Miranda Román, Alberto López Traba, Yeilen Aguilera, Abel Díaz Díaz, Azuquahe Pérez Hernández, Beatríz Báez Acosta, Daysi Chi

**Affiliations:** 1 Universidad de Las Palmas de Gran Canaria (ULPGC), Las Palmas de Gran Canaria, Spain; 2 Neurology Department, Hospital General de Fuerteventura, Canary Islands, Spain; 3 Pathology Department, Complejo Hospitalario Universitario Insular Materno-Infantil, Las Palmas de Gran Canaria, Spain; 4 Neurology Department, Complejo Hospitalario Universitario Insular Materno-Infantil, Las Palmas de Gran Canaria, Spain

**Keywords:** Oculoleptomeningeal amyloidosis, Transthyretin, Cerebral amyloid angiopathy, Transient focal neurological episodes, Leptomeningeal biopsy, Case report

## Abstract

Cerebral amyloid angiopathy (CAA) is most commonly associated with β-amyloid deposition, but other amyloid proteins may accumulate in leptomeningeal and superficial cortical vessels. We report a man first evaluated neurologically at 44 years of age after approximately one year of recurrent upper-limb and perioral sensory symptoms with speech disturbance, retrospectively considered transient focal neurological episodes (TFNEs). During follow-up, he developed bilateral vitritis and progressive behavioral, psychiatric, and cognitive impairment. Brain computed tomography showed diffuse linear and serpiginous leptomeningeal hyperdensities, consistent with calcified amyloid deposits with focal cortical subarachnoid hemorrhagic lesions SAH); angiography showed no underlying vascular abnormality. Follow-up neuroimaging demonstrated persistent leptomeningeal abnormalities and hemorrhagic lesions. Leptomeningeal biopsy revealed Congo red- and thioflavin S-positive vascular and meningeal amyloid deposits, with negative β-amyloid and amyloid A immunohistochemistry. A subcutaneous adipose tissue biopsy supported systemic amyloid deposition, and genetic testing identified the heterozygous TTR c.265T > C, p.(Tyr89His) variant. Electroneurography showed no large-fiber peripheral neuropathy. An off-label trial of tolcapone produced no clinical benefit. The patient subsequently developed severe cognitive and functional disability and progressive hydrocephalus, and died at 55 years of age from acute respiratory failure. This case highlights TTR-related cerebral amyloid angiopathy (TTR-CAA) as an important non-β-amyloid cause of TFNEs, calcified leptomeningeal infiltration, focal superficial hemorrhage, and progressive neuropsychiatric decline.

**Supplementary Information:**

The online version contains supplementary material available at https://doi.org/10.1007/s10072-026-09398-2.

## Introduction

CAA comprises a heterogeneous group of disorders characterized by amyloid deposition in leptomeningeal and cortical vessels. Although sporadic CAA is usually caused by β-amyloid, other amyloidogenic proteins can produce inherited or systemic forms of cerebral vascular amyloidosis [1, 7]. Correct identification of the deposited protein is clinically relevant because the associated systemic manifestations, inheritance pattern, prognosis, and therapeutic implications differ.

Hereditary transthyretin amyloidosis (ATTRv) usually affects the peripheral nerves and heart. However, transthyretin produced within the choroid plexus and ocular tissues may give rise to central nervous system (CNS) and ocular amyloid deposition. Rare TTR variants are associated with oculoleptomeningeal amyloidosis (OLMA), a phenotype characterized by vitreous involvement, leptomeningeal and superficial vascular amyloid deposition, transient focal neurological episodes (TFNEs), seizures, hemorrhagic or ischemic stroke, psychiatric manifestations, and progressive cognitive decline [1–3, 10].

We describe a histopathologically confirmed case of TTR-CAA associated with the TTR c.265T > C, p.(Tyr89His) variant. The case illustrates the diagnostic difficulty created by early psychiatric manifestations, initially unrevealing neuroimaging, absent peripheral neuropathy, and later meningeal and hemorrhagic findings.

## Case report

A 44-year-old man was first evaluated by Neurologybecause of recurrent transient neurological episodes that had begun approximately one year earlier. These consisted of numbness affecting either upper limb, sometimes accompanied by perioral numbness and difficulty articulating speech, and lasted approximately one hour. There was no lower-limb involvement or loss of consciousness, and the interictal neurological examination was normal. The episodes were initially attributed partly to emotional distress, although structural neurological disease was investigated. In retrospect, they were considered compatible with TFNEs. Medical records also documented intermittent persecutory delusional ideation initiated few years ago.

During follow-up, language arrest and alternating hemisensory or sensorimotor episodes persisted, while neurological examinations and brain CT remained unremarkable. Brain MRI and magnetic resonance angiography performed were reported as normal; the MRI protocol did not include a blood-sensitive sequence. The patient underwent surgery for bilateral vitritis, involving both age.

**Fig. 1:**
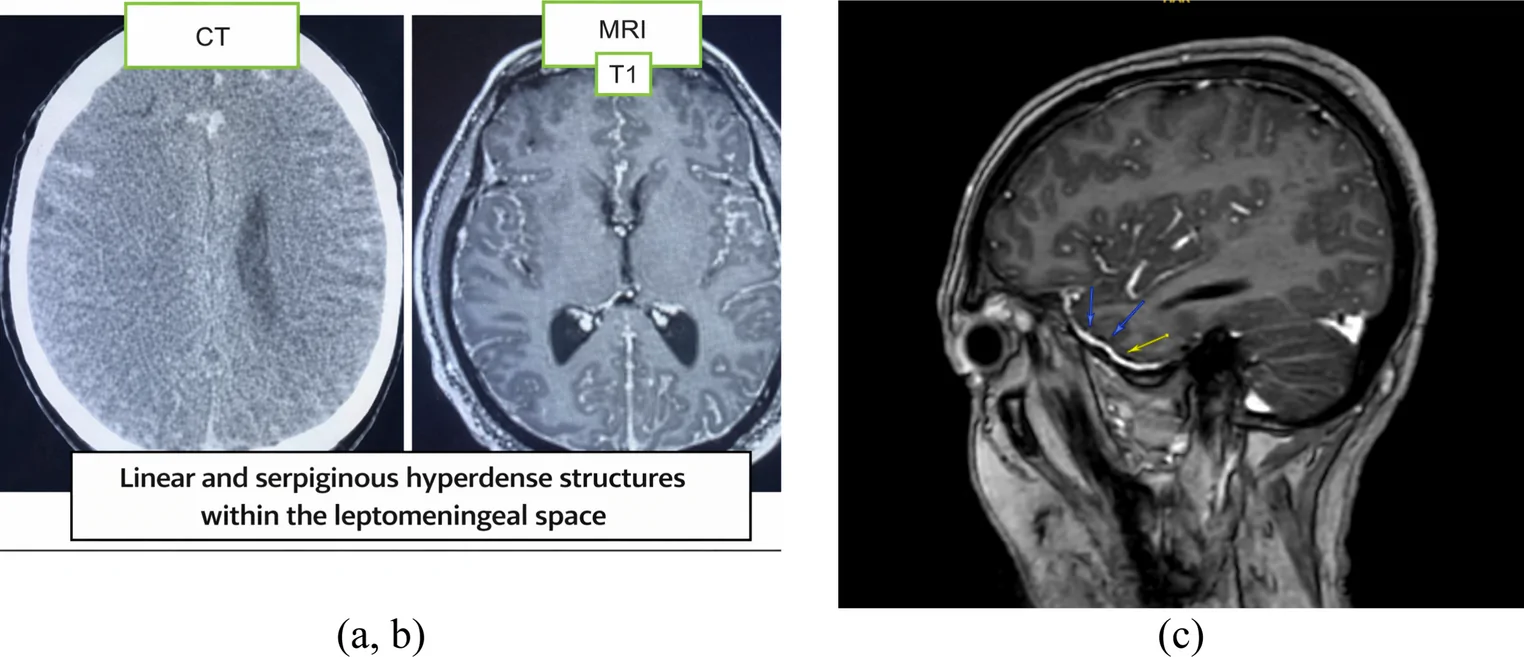
Neuroimaging findings. **a** Non-contrast CT showing diffuse linear and serpiginous leptomeningeal hyperdensities consistent with calcified amyloid deposits, with superimposed focal cortical hyperdensities compatible with subarachnoid hemorrhage. **b**, **c** Contrast-enhanced T1-weighted MRI demonstrating irregular leptomeningeal and pachymeningeal thickening and enhancement, predominantly involving cortical and subarachnoid regions

3 years later, at 47 years of age, he was admitted with headache, anxiety, and abrupt delusional behavior. CT demonstrated diffuse bilateral linear and serpiginous leptomeningeal hyperdensities, subsequently interpreted as calcified leptomeningeal amyloid deposits, together with superimposed focal cortical hyperdensities consistent with cortical subarachnoid hemorrhage. CT angiography and complete digital subtraction angiography showed no aneurysm, arteriovenous malformation, or other vascular abnormality. With further cognitive and psychiatric deterioration, follow-up CT showed persistent leptomeningeal hyperdensities, additional superficial SAH foci, and at least two subcentimeter intraparenchymal hemorrhagic lesions. MRI demonstrated irregular leptomeningeal and pachymeningeal enhancement. (Fig. 1).

At 49 years of age, neurological examination showed inattention, reduced and globally incoherent spontaneous speech, temporal disorientation, apraxia, and marked confabulation, without a persistent focal motor deficit. Cerebrospinal fluid contained 2 leukocytes/µL and 2 erythrocytes/µL, with glucose 88.8 mg/dL, protein 67.0 mg/dL, and mildly elevated lactate of 4.24 mmol/L. Extensive infectious, inflammatory, neoplastic, metabolic, and toxic investigations were unrevealing.

**Fig. 2:**
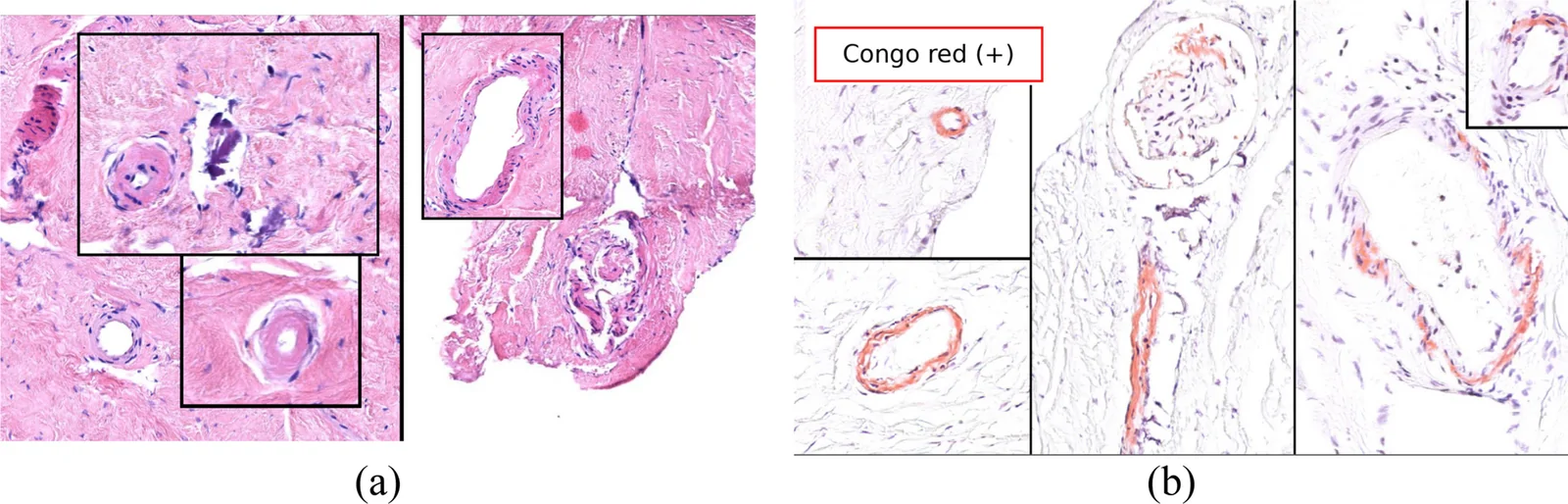
Histopathological confirmation of amyloid deposition in meningeal tissue. **a** Hematoxylin and eosin staining showing thickened leptomeninges and small- and medium-caliber vessels containing amorphous eosinophilic material within their walls. **b** Congo red staining showing vascular and leptomeningeal amyloid deposits

**Fig. 3:**
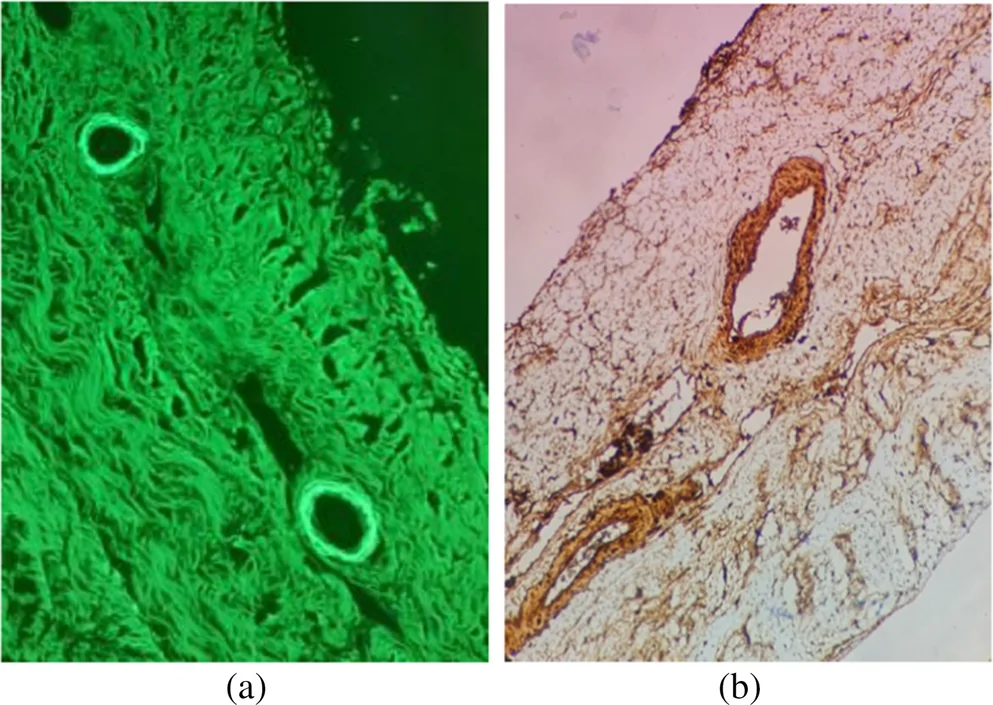
Amyloid confirmation and immunohistochemical exclusion of β-amyloid pathology. **a** Thioflavin S staining demonstrating amyloid deposition within vascular and leptomeningeal structures. **b** Immunohistochemistry for β-amyloid showing negative vascular and leptomeningeal deposits, excluding classical β-amyloid CAA

**Fig. 4:**
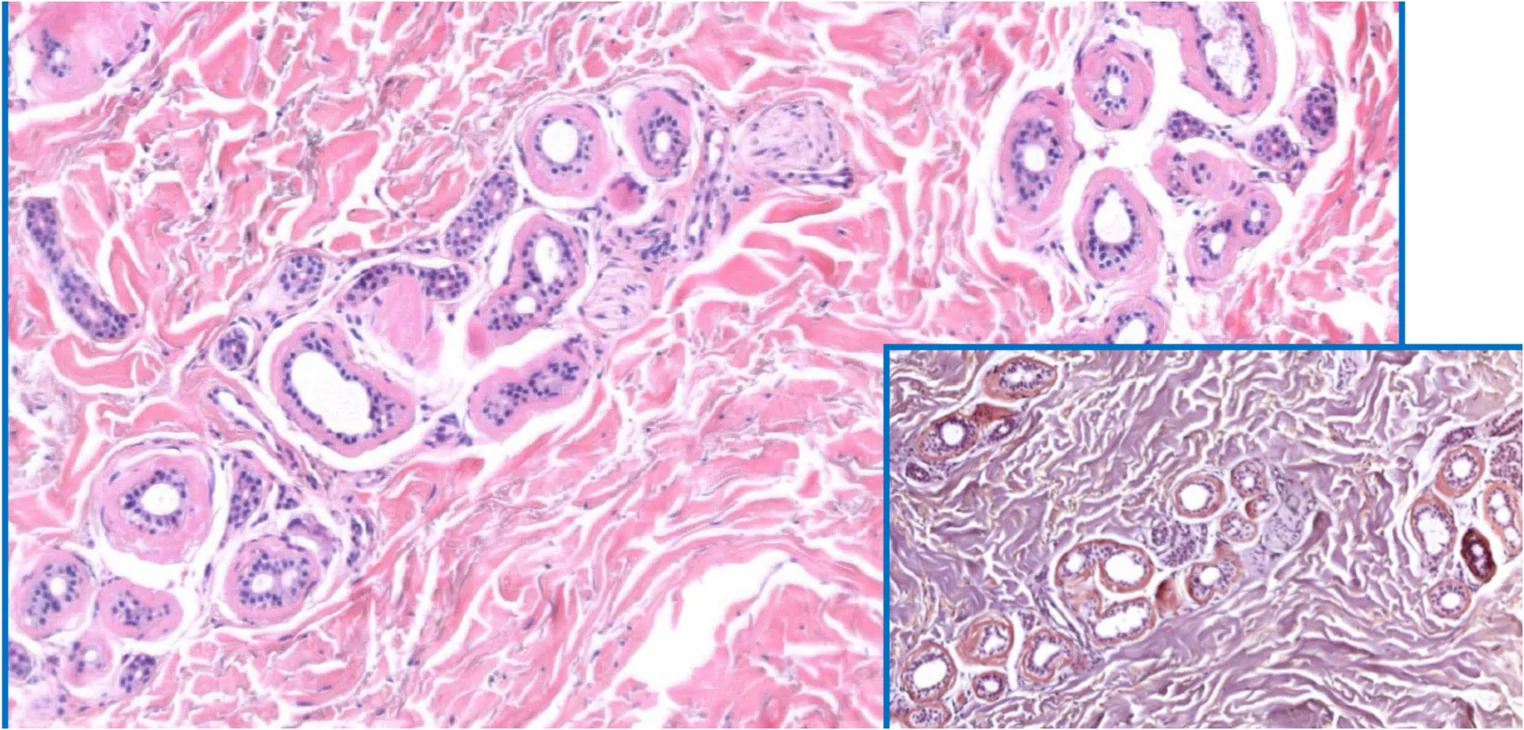
Evidence of extracerebral amyloid deposition. Congo red staining of a subcutaneous adipose tissue biopsy demonstrates amyloid deposition surrounding eccrine coils with minimal focal perivascular involvement

Because the progressive CNS-predominant syndrome and meningeal abnormalities remained unexplained, a leptomeningeal and cortico-subcortical biopsy was performed. Histological examination showed marked fibrosis, dystrophic calcification, and amorphous eosinophilic material within small-vessel walls. Congo red staining demonstrated apple-green birefringence under polarized light, and thioflavin S confirmed amyloid deposition. Immunohistochemistry was positive for amyloid P and negative for β-amyloid and amyloid A, with no light-chain restriction, inflammation, or malignancy (Figs. 2 and 3). A subcutaneous adipose tissue biopsy also showed focal Congo red-positive amyloid deposition surrounding eccrine coils and in occasional vessels, supporting extracerebral involvement (Fig. 4).

Genetic analysis identified the heterozygous TTR c.265T > C, p.(Tyr89His) variant, confirming hereditary transthyretin amyloidosis with predominant oculoleptomeningeal involvement.

Family screening identified a young adult daughter as a heterozygous carrier of the same pathogenic *TTR* variant (c.265T > C, p.Tyr89His). Family history revealed paternal psychiatric symptoms.

Electroneurography showed no evidence of large-fiber peripheral neuropathy. Transthoracic echocardiography showed preserved systolic function with a mildly thickened, granular-appearing interventricular septum.

Off-label tolcapone was started at 100 mg every 8 h with liver-function monitoring because of its ability to cross the blood-brain barrier and stabilize transthyretin. No clinical benefit was observed, and treatment was subsequently discontinued because of lack of efficacy and suspected adverse effects.

The patient developed progressive cognitive and functional deterioration, reaching a modified Rankin Scale score of 5 approximately four years after diagnosis. He became non-ambulatory and completely dependent for activities of daily living. Progressive ventriculomegaly accompanied by a clinical syndrome compatible with communicating hydrocephalus was subsequently documented. Its pathogenesis was considered probably multifactorial, in the context of extensive leptomeningeal amyloid involvement and previous cortical subarachnoid hemorrhagic lesions. Neurosurgical assessment concluded that ventriculoperitoneal shunting was unlikely to provide meaningful benefit because of the advanced neurological deterioration. While receiving palliative care, the patient developed acute respiratory failure and died at 55 years of age, with hereditary amyloidosis recorded as the underlying disease.

## Discussion

This case demonstrates that CAA is not synonymous with β-amyloid deposition. The combination of bilateral vitritis, recurrent TFNEs, calcified leptomeningeal infiltration with SAH foci, and progressive cognitive with psychiatric symptoms should raise suspicion of TTR-related CAA, particularly when conventional investigations do not support inflammatory, infectious, neoplastic, or structural vascular disease. Negative β-amyloid immunohistochemistry distinguished this disorder from classical CAA, while genetic testing established its hereditary TTR amyloidosis.

A less invasive biopsy may establish systemic amyloid deposition and should be preferred when it provides sufficient diagnostic information. In retrospect, TTR sequencing combined with an extracerebral biopsy could potentially have established hereditary ATTR without CNS sampling once this diagnosis was suspected. However, the patient initially presented with an unexplained CNS-predominant meningeal syndrome and an extensive differential diagnosis that included treatable inflammatory, infectious, and neoplastic disorders. The leptomeningeal biopsy directly demonstrated vascular and meningeal amyloid and excluded another pathological processes. The positive adipose tissue biopsy supported systemic involvement but, by itself, would not have established that the neurological syndrome resulted from cerebral amyloid deposition.

The natural history of OLMA is generally progressive, although the rate and sequence of manifestations vary. Ocular disease may precede or accompany neurological involvement. Reported CNS manifestations include headache, TFNEs, seizures, cognitive and psychiatric decline, ataxia, cranial neuropathies, ischemic or hemorrhagic stroke, superficial siderosis, and communicating hydrocephalus [1–3, 10]. The evolution in our patient from TFNEs and bilateral vitritis to calcified leptomeningeal infiltration with focal cortical subarachnoid, severe cognitive disability, hydrocephalus, and death illustrates this rapid progressive phenotype.

Hemorrhage is biologically plausible when amyloid infiltrates and weakens superficial vessel walls. A population-based study found that systemic amyloidosis was associated with an approximately threefold increased risk of intracranial hemorrhage, including intracerebral and subarachnoid hemorrhage [9]. Although this study did not distinguish between amyloid subtypes, its findings support an association between systemic amyloidosis and cerebrovascular fragility and are consistent with growing evidence that ATTR amyloidosis may involve not only peripheral organs but also the central nervous system.

Whether early recognition and treatment can modify the CNS natural history remains uncertain. Systemic ATTR therapies that stabilize circulating TTR or reduce hepatic TTR production may have limited effects on amyloid generated locally within the choroid plexus and ocular tissues [2, 4, 6]. Tolcapone crosses the blood-brain barrier and has shown experimental inhibition of aggregation and short-term stabilization of TTR in cerebrospinal fluid, but clinical efficacy against TTR-related CAA progression has not been established [11, 12]. No benefit was observed in this patient. Nevertheless, earlier diagnosis remains important for genetic counseling, family screening, and enrolment in future CNS-directed therapeutic studies.

The Boston criteria may recognize a CAA-like imaging pattern, but they cannot distinguish β-amyloid CAA from inherited angiopathies caused by other proteins, such as TTR [7]. Recent clinical series indicate that TTR-CAA can reproduce MRI features used in the Boston criteria [8]. When the phenotype includes ocular or systemic clues, relatively young onset, a suggestive family history, prominent leptomeningeal abnormalities, or an atypical course, amyloid typing and genetic analysis should be considered.

## Conclusions

TTR-CAA is a rare cause of TFNEs. Early MRI may be unrevealing, particularly when blood-sensitive sequences are absent. Extracerebral biopsy and genetic testing may establish ATTRv, whereas CNS tissue can be necessary when cerebral involvement remain uncertain. Accurate recognition enables genetic counseling and family screening and will become increasingly important as TTR CNS-directed treatments are developed.

## Supplementary Information

Below is the link to the electronic supplementary material.

Supplementary Figure S1. Additional histopathological images of the meningeal biopsy and Supplementary Figure S2. Clinical timeline of the index patient

## Data Availability

The clinical data supporting this case report are available from the corresponding author on reasonable request, subject to privacy and ethical restrictions.

## Abbreviations

TTR-CAA: Transthyretin cerebral amyloid angiopathy
OLMA: Oculoleptomeningeal amyloidosis
ATTRv: Hereditary variant transthyretin amyloidosis
TTR: Transthyretin
CNS: Central nervous system
CAA: Cerebral amyloid angiopathy
TFNE: Transient focal neurological episode
MRI: Magnetic resonance imaging
CT: Computed tomography
Aβ: β-amyloid
AA: Amyloid A
