# Supplementary material for: Beyond β-amyloid: transthyretin cerebral amyloid angiopathy
Source: Neurol Sci. 2026 Sep 26;47(10):816. doi: 10.1007/s10072-026-09398-2 (PMC13616015; doi:10.1007/s10072-026-09398-2)
Supplement: Supplementary file 1 [file 10072_2026_9398_MOESM1_ESM.docx]

**Supplementary material**

Supplementary Figure S1. Additional histopathological images of the meningeal biopsy.

Supplementary Figure S2. Clinical timeline of the index patient.
